# Devising a Distributed Co-Simulator for a Multi-UAV Network

**DOI:** 10.3390/s20216196

**Published:** 2020-10-30

**Authors:** Seongjoon Park, Woong Gyu La, Woonghee Lee, Hwangnam Kim

**Affiliations:** School of Electrical Engineering, Korea University, Seoul 02841, Korea; psj900918@korea.ac.kr (S.P.); juhgiyo@korea.ac.kr (W.G.L.); tgorevenge@korea.ac.kr (W.L.)

**Keywords:** multiple UAVs control, network simulator, UAV flight simulator, real-time infrastructure

## Abstract

Practical evaluation of the Unmanned Aerial Vehicle (UAV) network requires a lot of money to build experiment environments, which includes UAVs, network devices, flight controllers, and so on. To investigate the time-sensitivity of the multi-UAV network, the influence of the UAVs’ mobility should be precisely evaluated in the long term. Although there are some simulators for UAVs’ physical flight, there is no explicit scheme for simulating both the network environment and the flight environments simultaneously. In this paper, we propose a novel co-simulation scheme for the multiple UAVs network, which performs the flight simulation and the network simulation simultaneously. By considering the dependency between the flight status and networking situations of UAV, our work focuses on the consistency of simulation state through synchronization among simulation components. Furthermore, we extend our simulator to perform multiple scenarios by exploiting distributed manner. We verify our system with respect to the robustness of time management and propose some use cases which can be solely simulated by this.

## 1. Introduction

Unmanned Aerial Vehicles (UAVs) are being largely utilized in scientific or industrial applications. The main reason for the UAVs’ spotlight is their accessibility; UAVs can be operated in extreme environments where humans or conventional devices cannot be. Operating multiple UAVs that seek a common objective has been continuously addressed for decades [1,2], and recently spotlighted owing to the exploding availability of swarming 3D mobilities. For example, in a networking domain, several studies proposed instant network provision system by flying a swarm of drones establishing a multi-hop network, and deploying wireless access networks to provide the Internet to the terrestrial mobiles [3,4,5,6]. Based on this concept, we previously analyzed the traffic dynamics of drone-based network infrastructure and designed an algorithm that determines the formation of drones to maximize the network throughput [7]. Due to the proliferation of wireless communication devices and the blueprint of the Internet of Things (IoTs) shown in [8], network design aggressively contains UAV as major components and the researchers prepare the robust networking with UAVs [9]. In particular, achieving time-sensitive network (TSN) via UAVs has been recently spotlighted for instant but deterministic networking [10,11]. However, because of the spatial constraints to legitimately research or verify the fleet flight system and control mechanism, a number of experiments requiring expensive equipment (over 10 or more drones) or large areas have less chance to be conducted. As a result of this restriction, multi-UAV simulators [12,13,14,15,16,17] were proposed to model and simulate the motion of a fleet of UAVs.

One of the critical challenges about simulating the multiple UAVs flight originates from how to operate. As aforementioned, real-world UAVs maintain their fleet status by wireless networks, so the network performance of each UAV and its control efficiency are tightly related. The common method to simulate multi-UAV network while keeping the correlation between the flight control and the networking might be stepwise, which means that the operator conducts flight simulation first, saves the results, and then progresses the network simulation with the logged flight paths of UAVs. This method cannot run the network-induced UAV flight scenario, due to the dependency of the collective simulation sequence. Thus, this procedure is disadvantageous in terms of the simulation time, as well as the memory redundancy. Furthermore, if a certain issue having an effect on the UAVs’ flight is found during the network simulation, the expected movements of UAVs should be recalculated according to a modified scenario and then network simulation should be run again. It means, since most flight simulators do not consider the communication status of multiple UAVs, the common stepwise method is unable to simulate a number of network-dependent flight scenarios such as cooperative air fleet control [18,19] or time-sensitive networking [20].

To relax such limitation, we propose DUSC, a Distributed UAV-network Simulation Coordinator, which allows for running multiple simulation components on distinct agents simultaneously. Our key contribution is to design a system which guarantees reliability and real-time availability by integrating the existing simulators while considering that UAVs constantly change their position, and the change directly affects the performance of the wireless communication between the nodes. A simple method which runs both flight simulators and network simulators in parallel and exchanges the resulting information is unreliable, since the simulation time of each simulator runs differently and brings unmatched results. We address how to remedy this asynchronism by referring High-Level Architecture (HLA) design [21,22], especially its time management solution. The main consideration of time management is the dependency between the events—for example, network performance change by the UAVs movements or event-triggered communication and formation control [23,24]. We define these dependencies in two categories and propose a time management scheme for handling them. By exploiting input and output mechanisms of each simulator, DUSC can simplify its time management scheme while holding the synchronism. Our experiment and use cases show that DUSC results in the same output data with the collective method while the asynchronous method results in unreliable and skewed data. In addition, we show that the simulation time of DUSC is almost the same as the asynchronous method, which is 20% faster than the collective method.

In addition, we propose the DUSC network which describes how to run multiple scenarios simultaneously. DUSC can take advantage of the distributed computing environment where multiple machines are connected by wired or the wireless network because each simulator runs in parallel. We bring the server–client network model to design this system, exploiting the distinctive features of flight and network simulators.

To further verify the usability of DUSC, we introduce some use cases that can be simulated and studied by our system. First, we perform a comparison experiment of routing protocol in multi-UAV wireless networks. Since a multi-UAV network establishes a wireless ad hoc network, routing protocol used by the nodes directly affects the performance of the applications. We compare the overall network throughput with various routing protocols, such as Direct-Sequence Distance Vector (DSDV) [25], Optimized Link-State Routing (OLSR) [26], and Ad hoc On-demand Distance Vector (AODV) [27]. By leveraging the large scalability of DUSC, we conduct this comparative study while varying the number of UAVs and their network scenarios. Second, we designed a simple flight guidance system and simulate the system by using DUSC. A single or multiple UAVs are connected with multiple terrestrial nodes, and each UAV determines its direction from a number of terrestrial nodes’ direction guidances. This system requires the fine-grained synchronization between the network and the flight simulation, since the networking results in the flight control, so the network performance directly matters to the UAV’s flight path. We vary the density of terrestrial nodes and compare the results of simulations.

This paper is organized as follows: Section 2 summarizes related work on flight simulators and co-simulators, Section 3 introduces the DUSC architecture, and Section 4 addresses how to synchronize the time between simulators. Then, we address a DUSC network scheme in Section 5. Section 6 describes how DUSC and multi-DUSC system are implemented, and Section 7 verifies the consistency of the simulation results. Section 8 shows the use cases utilizing DUSC and Section 9 concludes the paper.

## 2. Related Work

The physical threats and high costs that can occur in operating multiple UAV systems are increasing the demand for flight simulation software [12]. The main purpose of the UAV flight simulator is to validate the system design, so the simulator should provide a number of features to port not only the physical engine, but also user-defined mechanisms into the environment. For doing so, most UAV simulators have interfaces to communicate with users, such as Heads-up Display (HUD), CLI or GUI, and so on. Specifically, some simulators [13,14,28,29] provide open source libraries or APIs for users to implement customized flight control modules or physical frames and utilize them for in-depth research. However, simulating networks of multiple UAVs is not included by default in traditional simulators because network simulation requires a large amount of resources to drive events from the physical layer to the application layer, which significantly slows the simulation speed. Instead of integration, our approach aims at coordinating separate simulators, which makes use of multi-core availability and improves the distribution of tasks over the network.

Network simulator implements a computer network and measures the system by simulating a specific scenario of networking [30]. Most network simulations require a heavy architecture because the network itself is made up of multiple layers and requires orchestration for them. Meanwhile, as UAV has been regarded as a participant of the promising networking node [31,32,33,34], verification and evaluation of UAV networking becomes a major topic of network simulation tools. To the best of our knowledge, there is no network simulator that internally implements the mobility of UAVs, and this circumstance comes to the motivation of our study.

The coordination of multiple simulators, also known as *co-simulation*, is aimed at parallel execution of the simulation environment. In addition to minimizing dependencies between simulators and running multiple events in parallel, this approach has a performance advantage over the collective method mentioned in Section 1, as it provides opportunities for multi-core execution or distributed computing [35]. Since the performance improvement is based on the parallel computing, co-simulation would take much less time than the collective method for longer or large-scale simulation. However, co-simulation has been a challenging topic for decades, mostly because of the time synchronization [36]. Since simulators secure their own time tables separately, their communication and consensus should be elaborately designed for accurate verification of the system. Furthermore, the progress of simulation time is necessarily variable. As the number of UAVs increases, the execution time allocated for the UAV flight simulator decreases because the network simulator’s completion time is delayed due to the busy traffic of the complex network topology (usually caused by the large network size). These discrepancies are not critical for the collective method, but co-simulation must synchronize the progress of the simulation time to ensure the reliability of the results while maintaining the benefits of co-simulation. We intensively address the time management issue of our proposed system in this paper.

## 3. Dusc Design

DUSC refers a co-simulation architecture that performs multi-UAV networking scenarios. We defined the parameter sharing scheme between the UAV flight simulator and the network simulator, from the fact that UAVs change their location in flight, and this changes network performance. It is notable that UAVs normally transmit the wireless signal in a less-obstacle medium, so the physical distance between UAVs dominantly takes effect on the quality of networking. Therefore, the network simulator should tightly track the flight path of UAVs. On the contrary, UAV control signals (packets) can be transmitted through the network, so the flight simulator should tightly monitor the network status. To preserve this dependency, we propose a reliable time-synchronized co-simulation scheme, following Run Time Infrastructure (RTI) protocol [21]. Section 3.1 overviews the entire design of DUSC, and Section 3.2, Section 3.3 and Section 3.4 address each component in detail.

### 3.1. System Design

**Figure 1:**
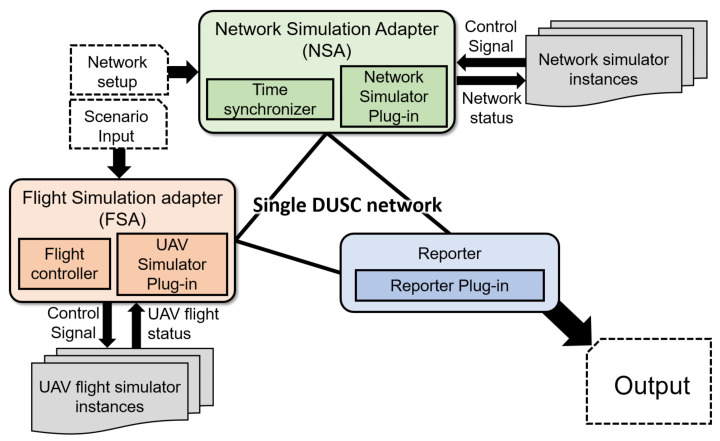
Architecture of DUSC.

DUSC is composed of three main parts: *Flight Simulation Adapter* (FSA), *Network Simulation Adapter* (NSA), and *Reporter* as shown in Figure 1. FSA manages a single or multiple instances of flight simulators while providing the interfaces of control algorithms given by a user. In addition, FSA exploits flight status that may affect the network simulator such as location or velocity from flight simulators and sends the status information to the NSA. NSA sets up the network environment such as network protocols and Tx/Rx power according to the configuration input established by a user or flight simulator, and runs a single or multiple instance of network simulators according to the environmental changes given by FSA. To synchronize the simulation time of two different simulators, NSA uses our novel time synchronization scheme (Section 4.1), which allows for simultaneously running network and flight simulators without a loss of data dependency. Finally, the Reporter contains a user-defined reporter module that exports the simulation output. The following subsections describe the structure of each subsystem in detail.

### 3.2. Flight Simulation Adapter (FSA)

FSA controls the overall flow of the flight simulation of multi-UAV scenarios, consisting of Flight Controller and UAV Simulator Plug-in. The Flight Controller interprets the scenario by parsing the script which contains a set of commands written by the user, then relays the command to UAV Simulator Plug-in, which works as an interface of the actual UAV flight simulator. The UAV Simulator plug-in can load a single or multiple flight simulator when the simulation begins. If an employed flight simulator can simulate only single UAV, *n* simulators and *n* plug-ins are required for simulating a multi-UAV scenario.

Location and state changes of flying UAVs are derived by the simulator engine, which considers the environmental factors such as turbulence and obstacles, and control factors such as user commands and physical features. Then, the Flight Controller retrieves UAVs’ states from the simulator and sends the packets adding a timestamp to the NSA through the network.

Note that UAV flight simulation and the simulation result depend on the parameters derived from the UAV’s physical frame and specifications (i.e., fixed-wing or multi rotor), the number of rotors, and its flight system components such as flight controller and flight software stack [12]. This means that a number of UAV models and flight controllers support their own flight simulators and Hardware In The Loop (HITL) or Software In The Loop (SITL) modules. In our implementation, to guarantee the objectivity, we used RotorS [14], a Gazebo [37]-based open source multi-UAV flight simulator. By customizing the sources in RotorS, users can simulate any flight control mechanism that they desire. Section 6 explains the implementation in detail.

### 3.3. Network Simulator Controller (NSA)

FSA can connect to NSA and simulate the network environment for receiving the current network status. Similar to FSA, NSA provides an interface to the network simulation instances. When FSA establishes network connection with NSA, it calls a NetworkSetup method to configure the network simulation environment based on numerical parameters such as network size (the number of nodes), UAVs’ initial position, and any parameters of UAV network (protocols, map obstacles, and so on).

NSA opens a thread for each FSA connection and generates an instance of network simulation. Each thread operates simulation by start() or stop() command, which are called by FSA. Between the start() and stop() call, NSA operates network simulator and reflects location changes of one or more UAVs sent from FSA through a Network Simulator Plug-in.

Due to the network changes leveraged by location changes, a network simulator must not precedently advance its simulation time because the UAVs’ location could be changed at that advancing time. The violation of this dependency invokes inconsistency and inaccuracy of simulation output. For instance, if a flight simulator runs simulation with 0.6x speed (100 s elapsed to simulate 60 s flight) and network simulator runs simulation with 1.2x speed (100 s elapsed to simulate 120 s network), due to the performance of each machine, any time-related metrics are calculated with 2x errors and network throughput could not be trusted. To unify the simulation timeline, we propose a time management algorithm which follows a RTI protocol time management [21] scheme to synchronize the time-sensitive information in parallel. The details will be explained in Section 6.

### 3.4. Reporter

The Reporter component connects with FSA and NSA, and provides a stream of simulation outputs to user. The user of the simulator can override or modify the report method, which is defined in a Reporter Plug-in module. When configuring the co-simulation, the user can define the report cycle and the format of the reporting log. FSA and NSA receive the user-made configuration, collect the simulation results from the simulator instance(s), and transmit to the reporter component with a designated format of log. Through this distributed design of each component, network traffic can also be divided into the reporter-FSA and reporter-NSA flows, which contributes the congestion avoidance of the DUSC traffic.

## 4. Time Management

Time synchronization among simulators is a historic issue [38]. Since we run two different simulators, the UAV simulator and network simulator, their simulation time processes differently. Time skew along multiple simulators could not be allowed when there is any dependency between the evaluation metrics and simulation parameters. We define two types of dependencies in multi-UAV network simulation when the network simulator and the flight simulator are separated.

- **Control-After-Network (CAN) Dependency.**
  The deployment of the wireless nodes highly affects the network performance, in terms of the medium quality and the network topology [39]. Since UAVs could change their location any time, their fluctuating network connection and numerical metrics must be simulated with pre-given location information. This means that the network simulator should process its simulation time from *t* to t+1 after it secures the information of location changes of nodes from *t* to t+1, determined by the flight simulator.
- **Network-After-Control (NAC) Dependency.**
  It is obvious that UAVs receive their flight command through wireless communication. Since network connection or throughput could change over any time, their control decision or input command must be simulated with pre-given network event information. This means that the flight simulator should process its simulation time from *t* to t+1 after it guarantees the flight commands arriving on the time range *t* to t+1 are fully secured, determined by the network simulator.

The essential objective of the flight simulator is to control the movement of the UAVs. Unless the flight simulation scenario only contains a set of commands that hover over all of the UAVs, CAN dependency is applied in most of the applications. However, a matter of *where* to move UAVs is not always determined by network situation, depending on the application. Thus, NAC dependency could be relaxed by the assumptions that UAVs can always receive network-independent flight commands (by an internal path-planning system) or flight commands that are propagated in a sparse cycle. Furthermore, it can be removed in the circumstances where multiple UAVs’ movements are not related to the network. Trying to tightly keep both dependencies must incur deadlock, so we focus on CAN dependency first and loosen NAC dependency with a reasonable solution.

### 4.1. DUSC Time Management Algorithm

When using multiple UAVs, their simulation data streams should be observed. If a single flight simulator is used, location changes of multiple UAVs are collectively calculated in each cycle. Otherwise, if two or more flight simulators are used, their location changes are calculated separately in their own cycle. In addition, if each flight event is sent through reliable transport layer such as TCP, the events might be ordered for each UAV, but not globally in multiple UAVs. This means that the events might be transmitted in order, but not uniformly over all UAVs with respect to the time. By exploiting those observations, we propose a novel real-time synchronizing algorithm named the DUSC Time Management Algorithm (DTMA) to efficiently select the events that satisfy the above conditions, and run the simulation with selected events.

To achieve the time synchronism, we brought one of the standardized time management mechanisms from IEEE 1516 [21], which describes how to model and design the combination of simulation interfaces. Note that we specifically refer to the time management mechanism of IEEE 1516.1, not the entire architecture of HLA due to the difference of the global structure. HLA is formed as a centralized topology, where RTI manages a set of simulators, called *Federate*. However, DUSC is formed as the distributed topology where NSA and FSA directly share the simulation data to simulate a unified environment. We considered DTMA to guarantee the time synchronization in a distributed manner of a DUSC system, borrowing the design concept of HLA. RTI provides two types of time management modes: Time Advancing Request (TAR) mode (Section 8.8 of IEEE 1516.1) and Next Message Request (NMR) mode (Section 8.10 of IEEE 1516.1) [21].

Since the simulation data of flight simulation is not discreetly obtained, NMR is not suitable for DUSC. Referring to the service design of TAR, DTMA periodically informs NSA and FSA whether they should advance their time or not.

**Figure 2:**
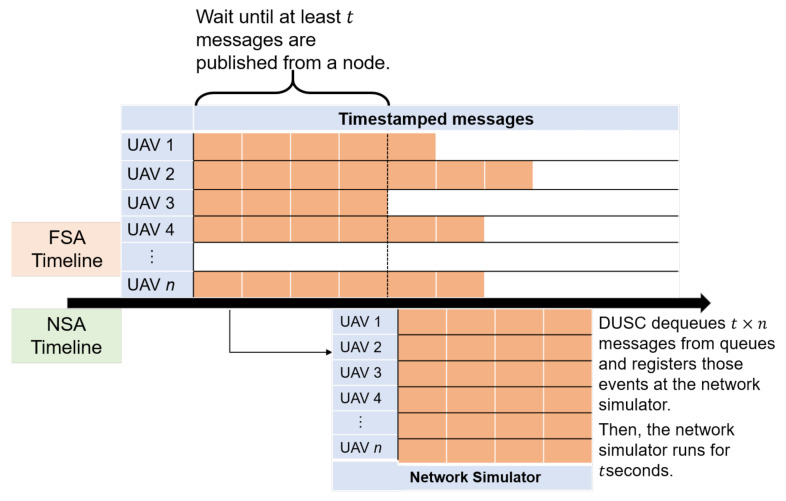
DUSC Time Management Algorithm (DTMA).

The overall sequence of DTMA is presented in Algorithm 1. At first, we set the *Time Advancing Granularity* to τ, which refers to a unit of time advance, which is represented by the number of the location events of a UAV. If the flight simulator updates UAVs’ locations in 50 Hz, the amount of time advance that can be calculated by τ/50 seconds. Until all *n* UAVs receive at least τ packets each, the system categorizes and enqueues the n×τ packets to the queues. Note that UAV location message transmission pattern can be varied depending on the flight simulator design. This means that single or multiple TCP sockets might be used to transmit multi-UAV information, and multiple sockets might send messages synchronously or asynchronously. We assume the most hostile situations where multiple sockets of FSA send the location of each UAV asynchronously. After the reception, DTMA grabs the location change from $n\times \min\left(N\left(S_{i}\right)\right)$ packets’ contents, which refers to the sequences of the UAV location changes that arrived entirely for all UAVs in a certain time. DTMA forwards these packets to Network Simulator Plug-in and dequeues them from each queue. Otherwise, DTMA should wait until n×τ packets are received since the next location change of each UAV is calculated at a flight simulator after the network simulator finishes. Enqueue is continued until the simulation ends. On the implementation, two threads run in parallel to operate network simulation and process the receiving packets simultaneously. Figure 2 shows a graphical representation of DTMA.

**Algorithm 1** DUSC Time Management Algorithm**Input:** Incoming packets *p*, the number of UAVs *n*
1:Create a set of queues $S=\{S_{1},S_{2},...,S_{n}\}$2:Set *Time Advancing Granularity* τ3:
**while Stop event is not raised do**
4:    Parse *p* and find UAV id *i* and location *l*5:    Enqueue *l* in $S_{i}$6:    **if** Network simulator is not started **or** NAC holds **then**7:        **if** $\min N(S_{i})>\tau$ **then**8:           Schedule τ locations for all UAVs9:           Dequeue scheduled values10:           Start network simulator for τ11:        **end if**12:    **else**13:        **if** Network simulator is not running **then**14:           Schedule $\min N(S_{i})$ locations for all UAVs15:           Dequeue scheduled values16:           Advance network simulator for τ17:        **end if**18:    **end if**19:
**end while**



### 4.2. Handling NAC Dependency

To comply NAC dependency, network simulation logs need to be shared with flight simulation. Reporting a whole network-related simulation log and parsing each message might cause the ambiguity of data domain and increase control decision time. To minimize the overhead, DUSC defines a RegisterNetworkCallback() method that makes FSA receive a packet composed of a certain network condition and additional simulation data when NSA satisfies such condition.FSA sends a RegisterNetworkCallback() command to the NSA, and NSA registers the condition of this command and monitors whether its network simulator module meets the condition. If the network simulator satisfies one of condition registered at NSA, it sends a callback message to FSA, and FSA operates the flight command scripted in the user-defined callback function.

### 4.3. DTMA Analysis

If NAC dependency does not hold, the τ value is initialized when a scenario sets up. The value of τ roughly affects the time advancing cycle after initialization. If τ is relatively small, the network simulator grabs a few number of flight schedules since the network simulator runs for a relatively small amount of time. On the other hand, if τ is relatively large, the network simulator grabs a large number of the flight schedules since the network simulator runs for a relatively large amount of time. Assuming that the time taken to resume or stop network simulation is negligible, total simulation time is almost constant regardless of the size of τ. Meanwhile, if NAC holds, the variation of τ causes a trade-off between the accuracy of synchronism and the total simulation time. A relatively small value of τ causes more control signals between FSA and NSA, so the system overhead might be increased. In the opposite case, the network overhead might be reduced, but the NAC dependency might be lost because FSA receives more delayed callback events, whose amount is the maximum τ. The minimum response time of the UAVs for the certain network event can be varied by users, so this paper does not propose which τ value is the best. In our implementation, τ is set to 5 since this value results in the most accurate and fast simulation on our simulation environments.

## 5. DUSC Network

**Figure 3:**
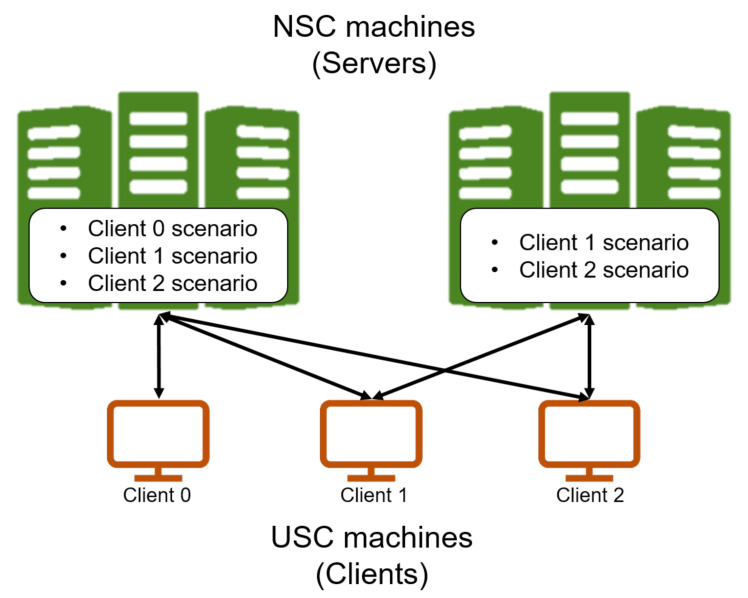
Distributed network of DUSC.

Each component of DUSC can be run either in the same machine or different machines while being connected by the network. This section presents the design of a multi co-simulation scenario by a distributed network as shown in Figure 3. If there is no NAC dependency (Section 4.2), broadcasting the locations of UAVs to multiple NSAs allows for simulating the various network environments in parallel with the same flight scenario.Otherwise, a specific communication between UAVs affects the motion of the certain UAVs, so there is bidirectional dependency between simulators and only one NSA can be matched. For instance, let us assume an application that a number of UAVs fly around a certain area while streaming their video recordings to the Ground Control Station (GCS). If UAVs’ flight schedule is determined by a certain status independent of the network status (i.e., remote control of the users), the performance can be simulated in a number of NSAs while varying the network parameters. However, if the UAVs’ flight is controlled by the network of themselves, such as a heterogeneous network architecture with sensor networks [34], multiple NSAs cannot be deployed since each NSA sends its network events differently to FSA depending on both the network configuration and the network status of a multi-DUSC system. This means that FSA should grab the network response observed from the NSA to control the UAVs, but the same sort of network events arrive from multiple NSAs in scattered time, which cause the unreliable results of the multi-UAV flight simulation. In this case, cloning an instance of a flight scenario and matching each to an NSA can be the solution. The DUSC network determines whether multiple NSAs can be matched or not by a Boolean-type variable, which is determined by the existence of RegisterNetworkCallback definition.

Finally, we exploit a server–client model to efficiently operate a DUSC network. To grant the real-time control availability to the flight simulation, we let the FSA and Reporter act as a client and NSA as a server, respectively. NSA listens to the network socket and creates a thread running network simulation when FSA requests the network connection. As receiving the simulation profile such as the number of UAVs and network configuration, a network simulator creates a set of nodes and constructs a network environment. By this approach, NSA can concurrently accept multiple FSAs’ requests, and multiple FSAs can perform their network simulation in multiple channels simultaneously.

## 6. Implementation

This section briefly introduces how we implemented DUSC. Note that the scope of DUSC does not include the design of actual network or flight simulators (Figure 1). We implemented NSA, FSA, and Reporter in Python, and the user command script was simply designed while comprising basic UAV flight commands, such as takeoff, land, waypoint, and so on. TCP connection and socket managing methods are provided by PyServer [40], an asynchronous TCP/UDP server/client library developed in Python.

Note that DUSC can be implemented as long as a simulator plug-in module can be made (Section 3). This means that simulators to be used should provide an API for running the backend engine. However, it is not available for any simulator since some do not authorize the access of resulting data to an exterior module, following their policies. Through the survey, we found a network simulator and multi-UAV flight simulator that satisfy the precondition of DUSC implementation while keeping the objectivity, which are Network Simulator 3 (ns-3) [41] and RotorS [14], respectively. Ns-3 provides a script-based environment for collecting the simulation data and operating the simulation status by utilizing its libraries. RotorS periodically transmits its simulation data and receives the operation command of the simulation from the user-accessible interface, called the Robot Operating System (ROS) [42]. The following subsections explain the implementation of each component of DUSC in detail.

### 6.1. UAV Simulator Controller (FSA)

Since RotorS provides its status through *ROS Topic*, FSA utilizes ROS API. The UAV Simulator Plug-in subscribes the topics of all UAVs’ statuses and FSA sends these to the NSA through the DUSC network. The Simulator Controller publishes the command flight topics of each UAV in RotorS, by parsing the user command. The user command includes the whole simulation control messages, such as start(), stop(), and pause(), and the flight control messages such as takeoff(), land(), waypoint(), and RegisterCallback() (Section 4.2). The flight simulation profile including the number of UAVs or their HW/SW model is written in Extensible Markup Language (XML), following the Gazebo platform.

### 6.2. Network Simulator Controller (NSA)

Ns-3 provides its network simulation library written in C++, and developers can use the library by writing scripts in C++ or Python. Network Simulator Plug-in configures a network environment following the profile received from FSA and forwards it to the network simulator. The time synchronizer runs DTMA (Algorithm 1) by receiving packets through the DUSC network and hands over the synchronized schedule to a Network Simulator Plug-in. The Network Simulator Plug-in periodically decides whether it should send a callback event to FSA or not, by checking the conditions which are registered by RegisterNetworkCallback() command.

### 6.3. Reporter

Reporter Plug-in streams the status of UAV flight and their networking received through a network via specific output devices or files. Storing and managing data of Reporter component is entirely user-defined. In our experiments, Reporter writes data logs in files for analysis.

**Figure 4:**
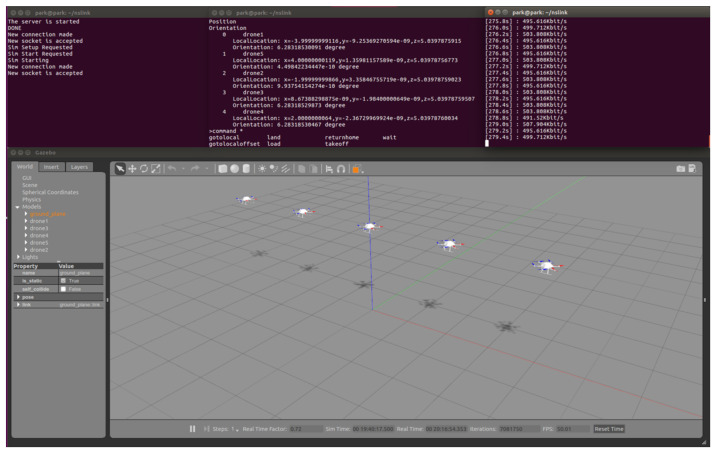
Implementation of DUSC.

Figure 4 shows the implementation result of DUSC on Ubuntu 16.04. Five drones were arranged in a line with a 2-m distance interval, and we wrote a simple network scenario where the drones transmit packets to a terrestrial sink node located in (0,0,0) with 500 Kbit/s data rate each. To show the implementation of DUSC in a screen, all components were executed in a single PC. The top-left terminal runs NSA (Section 3.3) and it acted as a TCP server (Section 5) establishing connection with the other DUSC components. The top-middle terminal runs FSA (Section 3.2). By taking in the user command or script, FSA forwarded the command to the bottom window, which is operating the RotorS [14] simulator. With the takeoff() command, drones took off at the altitude of 2 m as shown in the bottom window. The top-right terminal runs Reporter (Section 3.4) and periodically outputs the throughput of a sink node.

## 7. Experiment

We compared our system with two other co-simulation methods to prove the effectiveness of the DUSC system:

1. **Non-Real-Time**: a traditional stepwise method that records UAVs’ flight positions and then runs the network simulator utilizing a recorded flight log.
2. **Asynchronous**: a simple parallel method that runs both simulators simultaneously without any time synchronization algorithm.

**Figure 5:**
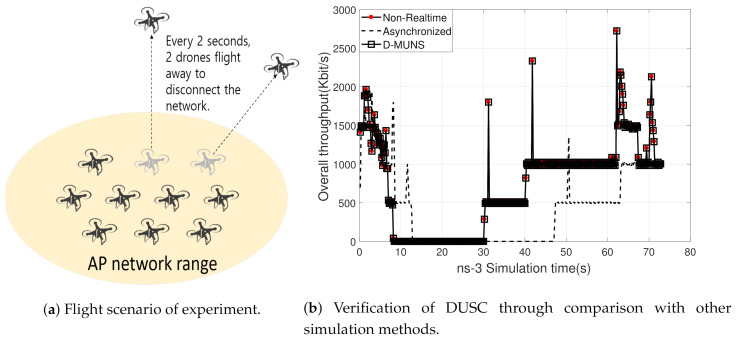
DUSC verification.

Multi-UAV’s flight scenario was written as a script and Figure 5a shows this scenario graphically. The flight scenario was sequenced as:

1. 10 UAVs are gathered around nearby origin (0,0,0) and takes off at 3.0 m above.
2. Every two seconds, two UAVs are scattered far enough apart to lose the network connection from the others.
3. After all UAVs are scattered, two UAVs come back to their original location every 10 s.

The UAVs transmit a chunk of data to the sink node with 0.5 Mbps, and the Network simulator measures the aggregated throughput of UAVs at 5 Hz and reports to the Reporter module through the DUSC network.

Figure 5b shows the measured throughput with respect to the time for each method. As shown in the figure, DUSC showed exactly the same result with a Non Real-Time method while the Asynchronous method showed the time-delayed result due to the processing time of the UAV simulator. This result proves that DUSC stably synchronizes the time values of distinct simulators in parallel operation while distributing each component into the different machines.

In addition to the simulation result, we measured the total simulation time of each method. Total simulation time is defined as the elapsed time from the start of any simulator operated for the experiment to the end of all simulators. The average time required for each method is 156.7 s (127.4 s for flight simulation and 29.28 s for network simulation) for the Non-Real-Time method and 129.2 s for Asynchronous and DUSC. In conclusion, DUSC reduced the simulation time by 17.5% in this scenario, while robustly performing the synchronized co-simulation.

Because the simulation instances of DUSC run in parallel, total simulation time refers to the time for all instances to finish their simulation. If the number of UAVs increases, flight simulation time increases, and network simulation time also does if all UAVs raise network events. If the networking scenario is only changed to run heavier traffic, the network simulator mostly takes more time than a flight simulator. The main reason for the DUSC’s performance improvement comes from the parallelism, which reduces total simulation time to the maximum simulation time of components. Let $T_{F}$ and $T_{N}$ refer to the required time for flight simulation and network simulation when solely executed, respectively. The Non-Real-Time method would take the total required time of $T_{F}+T_{N}$, except for the intermediate process finalization time and initiation time. In the case of DUSC, total required time can be approximated to $\max(T_{F},T_{N})$, since the faster one waits for the other simulation to synchronously advance its time table. The empirical performance gap (1.8 s of experiment) is by the networking delay between the simulation instances and the control overhead invoked by pause/resume signals. In conclusion, the expected performance gain of DUSC compared with the Non-Real-Time method is $\approx 1-\left(\max\left(T_{F},T_{N}\right)\right)/\left(T_{F}+T_{N}\right)$, which proportionally increases as the simulation configuration and simulation execution time increase.

## 8. Use Cases

Since the time synchronism of DUSC is verified, we introduce some use cases that utilize DUSC to evaluate the multi-UAV network systems. One case addressed in Section 8.1 only has CAN dependency, and the other case addressed in Section 8.2 has both dependencies. In the following subsections, we explained simulation experiments in detail.

### 8.1. Routing Protocol Comparison

**Figure 6:**
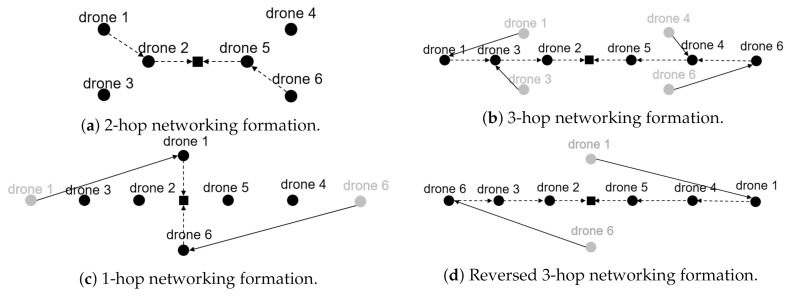
Fleet formation of multi-UAV network.

**Figure 7:**
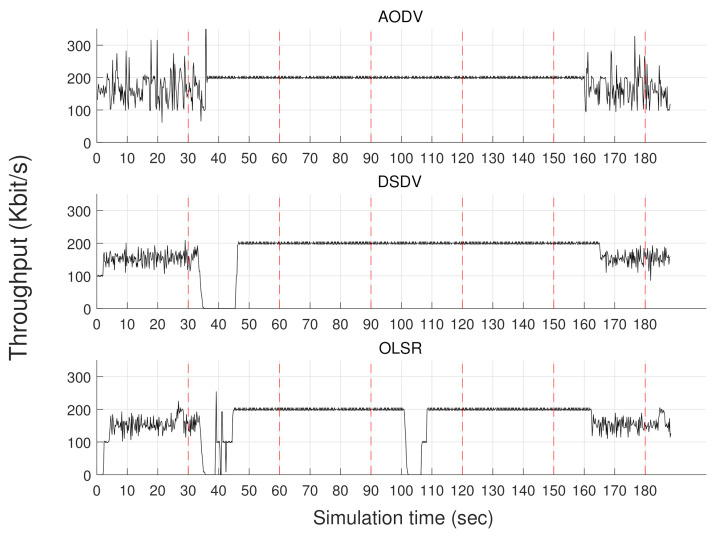
Network throughput comparison of routing protocols.

We compare the widely-used wireless routing protocols on the multi-UAV’s network environment via DUSC. We simulated DSDV [25], OLSR [26], and AODV [27], which are the routing protocols that multi-UAV can use. The simple flight and network communication scenario is graphically represented at Figure 6. At first, six UAVs are hovering with the formation as shown in Figure 6a. Then, every 30 s, UAVs sequentially change from the formation as shown in Figure 6b–d. Finally, at the formation of Figure 6d, UAVs sequentially change back into the formation of Figure 6a–c every 30 s. Following the flight scenario, two UAVs indicated as drone 1 and drone 6 send packets to the sink node located at the origin with a 100 Kbit/s data rate. For each formation, the expected routing paths of these two UAVs are graphically expressed as dotted lines, and the overall UAVs’ flight paths are represented as solid lines in Figure 6. By configuring the network environment such as the wireless transmission power, we assure that drone 1 and drone 6 cannot send packets in one hop except for the case of Figure 6c. The overall network throughput with respect to the time is shown in Figure 7.

Our simulation result can be analyzed in two-folded ways:

- **Selection of the routing protocol.** As shown in Figure 7, the network throughput of AODV outperforms the other results, especially when the formation is changed. Thus, the AODV protocol can be the most appropriate solution for multiple UAVs among the presented routing protocols.
- **Network-robust formation strategy.** The result shows that network throughput is relatively stable from 60 s to 150 s. Figure 6c,d show the formations of UAVs during that time. Hence, although the hop count is increased, the UAVs in the in-lined formation such as Figure 6b,d provide a more robust network quality than the UAVs in the formation shown in Figure 6a, which has a contention. While scheduling the flight scenario of the multiple UAVs, DUSC can simulate the network quality of the UAVs with respect to their formation or moving paths.

Since the optimization of the multi-UAV’s formation or routing protocol is out of the scope of this paper, we leave the research on cases where the UAVs are in more complicated formations as future work.

### 8.2. UAV Flight Guidance System

We introduce a simple flight guidance system which lets terrestrial network nodes cooperatively guide a single or multiple UAVs. As [43] already presented, the Ground Positioning System (GPS) might be insecure if attackers falsify global location information. In the case in which a UAV cannot trust its GPS device, we considered a possibility to obtain the correct direction by requesting to the nearby nodes. Assuming that the stations on the ground already know their locations, they can guide the movement of nearby UAVs. At this time, since UAV and the stations are at different locations, their guidance is not accurate, proportional to the relative distance betwee n the UAV and a station. Thus, the UAV should set the direction by the weighted sum of the guidances.

**Figure 8:**
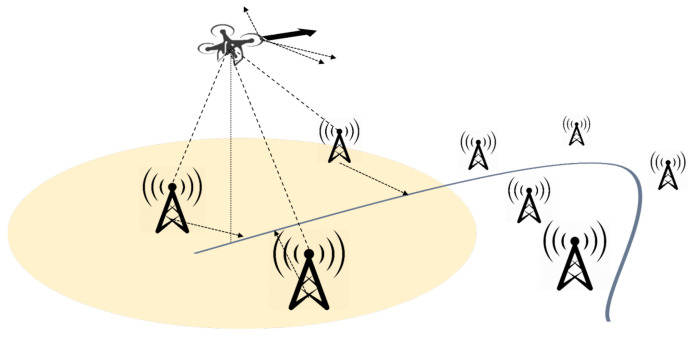
A simple UAV flight guidance system.

We designed the guidance system that constructs the network by letting each UAV and each terrestrial node act as an AP and a mobile station, respectively, shown as Figure 8. During the flight, the UAV periodically broadcasts its flight schedule (a set of all waypoints) $P=\{\vec{p_{0}},\vec{p_{1}},\vec{p_{2}},...\}$, expressed in latitude and longitude. Terrestrial network nodes near the UAV receive this schedule and calculate a *guidance* vector, referred to as $\vec{g}$, which states what direction UAV chooses to fulfill in the flight schedule. A terrestrial node finds the nearest waypoint $\vec{p_{m}}$ from its position in the flight schedule, and calculates the vector from the position as $\vec{g}=\vec{p_{m+k}}-\vec{p_{n}}$, where a positive integer *k* refers to the *guidance resolution*, and $\vec{p_{n}}$ refers to the node’s position. Then, the UAV determines its direction by calculating

(1) $$D=\sum_{i\in N}w_{i}\vec{g_{i}}$$

where $w_{i}$ refers to a scalar value indicating the weight, inversely proportional to the relative distance. If the terrestrial node is close to the last waypoint of the UAV, the node does not calculate the vector since the UAV seems to arrive. The UAV periodically repeats the above sequence—broadcast, guidance collection, and direction decision—until all nearby terrestrial nodes do not give guidance messages.

**Figure 9:**
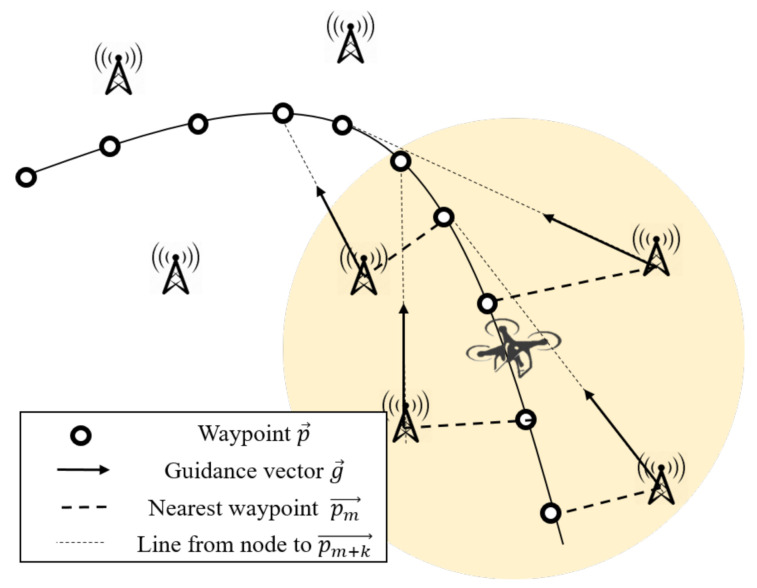
Collected guidance vectors from nearby APs.

Figure 9 graphically represents how the guidance vector is calculated. In the figure, a yellow circular space represents the approximated communication range of the UAV. As can be seen from the generated $\vec{g}$, the guidance errors that occurred from the nodes located on both sides of the UAV are canceled after the summation of the vectors. However, if a number of nodes are densely located on a single side of the UAV, a result direction can be biased and the UAV can severely abandon the path. Therefore, we deployed the terrestrial nodes on the grid pattern in our simulation.

The primary goal of this guidance system is to verify the performance of DUSC, but this design has advantages from the following point of view:

- **Lightweight sensors**. Without a GPS device, UAV can determine its global direction through a network. Although global orientation should be obtained by some sensors such as magnetometer, this relaxation highly drops the operation cost.
- **Indoor/outdoor extension**. Substituting $\vec{p_{i}}$ to local frame operates the guidance system without difficulty. If using UWB, relative distance between UAV and the terrestrial nodes can be accurate, which refines $w_{i}$ to the precise one.
- **Portability with UAV traffic management (UTM)**. Research on UTM is eagerly addressed with the exploding demands on the stabilized drone management scheme [44]. Our station-based guidance system basically guarantees high scalability because any number of UAVs can be guided as long as the network resource and station resource allow them. This feature is what UTM primarily desires, so our guidance mechanism can be utilized to implement a large-scale UAV navigation system.

In order to maintain the context of this paper, we leave in-depth studies and analysis of the guidance system and an evaluation of its availability as future work.

We examined this guidance system through DUSC and measured the resulting track of UAV while varying the density of terrestrial nodes. The simulation of UAV guidance system holds NAC dependency, since a set of network nodes is defined by the network simulator. We utilized RegisterNetworkCallback() before starting simulation to track the link connection and disconnection between the UAV and terrestrial nodes. Positioning terrestrial nodes also affects the result of simulation, so we located n×n nodes on the grid with distance interval *I* and collected the track of the UAV while varying *I*. In addition, we added Gaussian distributed noise to the UAV’s actual direction to imitate the error of inertial sensors.

**Figure 10:**
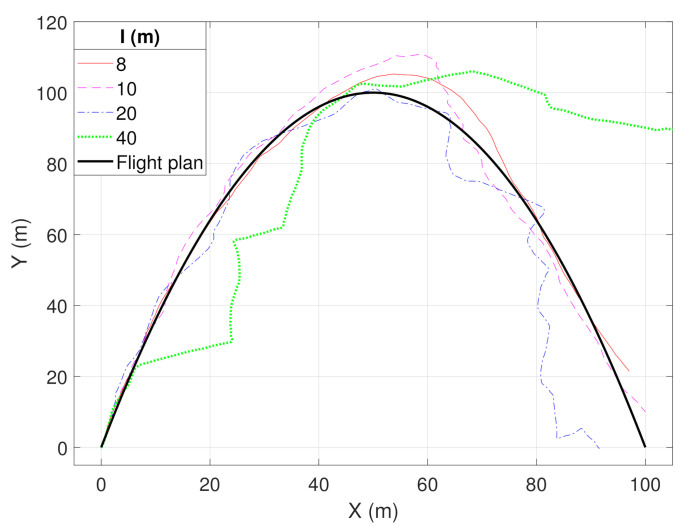
Simulation results of a UAV flight guidance system.

Figure 10 shows the result of this simulation. The expected flight path is shaped as a quadratic line following $y=-\frac{1}{25}x^{2}+4x$. The UAV starts flying with our guidance system at (0,0), and ends flying at (100,0). The actual flight track is collected by simulated odometry of RotorS, with no error. As the granularity of terrestrial nodes increases, terrestrial nodes guide the UAV to more accurate directions. Therefore, the most accurate flight track can be obtained when I=8. Otherwise, when I=40, the UAV completely lost its direction after x=50 due to the lack of guidance. The results show that DUSC can reliably simulate various systems and the research of the multi-UAV network.

## 9. Conclusions

We designed a co-simulation system which concurrently simulates the flight and the network environment of the multiple UAVs. The overall contents of this paper can be summarized as follows:

- We proposed DUSC, which controls the existing simulators and combines the information of them.
- We addressed the dependencies between the flight and the network simulators while simulating the multiple UAVs’ network, and established the strategy to keep these dependencies.
- To achieve the time synchronism between the simulators, we brought the concept of HLA time management design for the adaptive time-advancing control of the simulators.
- By exploiting the distributed design of DUSC, we proposed the DUSC network to simultaneously run the multiple simulations in the various configurations and environments.
- We implemented our DUSC design and verified the consistency of simulation data through the experiment.
- We proposed some use cases to be simulated by utilizing DUSC, and showed that DUSC can be used to evaluate the performance of these cases.

DUSC is a collection of the simulator plug-ins and the data management modules, which does not entirely tune or modify the existing simulators. Such independence can be leveraged to the extensibility of the DUSC system, especially in terms of the complex combination of various simulators. This means that, not only limited to the network simulator, DUSC can combine user-requiring simulators such as an audio simulator or a weather simulator. In addition, time-wise multi-UAV formation algorithms that implement the swarming [1,7,45] can join as an adaptor of DUSC to replace the user’s handmade flight script. Additional dependencies induced by various kinds of the simulators and an abstracted time synchronization algorithm for the multiple agents should be discussed. In addition, in a multi DUSC system, an unknown number of clients (FSAs) are connected to the one or more servers (NSAs), which could cause the potential unbalance of the workload assigned to each server. Deploying a managing module that matches the clients to the specific servers while monitoring the available computation resources of all of the servers and optimizing the expected running time of multiple simulations will be left for future work.

DUSC aims to be the empirical tool for researching the wide possibilities of multiple drones as well as their network. Our motivation of the DUSC design originates from the practical tendency where the research utilizing multiple drones could cost an enormous amount of economic, time, and human resources, and the fact that there was no such integrated simulation system for multiple drones’ networking applications. By exploiting the simulator-independent aspect of this system, we expect that an unbounded range of multi-UAV missions can be simulated by the following research.

## Abbreviations

The following abbreviations are used in this manuscript:

UAV	Unmanned Aerial Vehicle
TCP	Transmission Control Protocol
UTM	UAV Traffic Management
